# Dancing with a seizure, a case report

**DOI:** 10.1186/s12883-017-0797-2

**Published:** 2017-01-25

**Authors:** Keun Tae Kim, Kon Chu, Sang Kun Lee

**Affiliations:** Department of Neurology, Comprehensive Epilepsy Center, Seoul National University Hospital, 101 Daehangno, Jongno-gu, Seoul Republic of Korea

**Keywords:** Dancing, Seizure, Semiology

## Abstract

**Background:**

Dancing is a very rare seizure semiology, and has only few case reports so far. Moreover, no case regarded as dancing with both description and video was presented.

**Case presentation:**

A 42-year-old woman with medical intractable epilepsy showed a typical semiology of right temporal lobe epilepsy: right hand automatism and ictal speech. The following semiology, appeared during ictal and post-ictal stage, was complex, rhythmical and sequential movement. It was enough to be called dancing.

**Conclusions:**

We hereby report the most plausible dancing in the ictal and post-ictal state, documented by simultaneous video and electroencephalography.

**Electronic supplementary material:**

The online version of this article (doi:10.1186/s12883-017-0797-2) contains supplementary material, which is available to authorized users.

## Background

Dancing is a very rare seizure semiology. To the best of our knowledge, there have been only a few case reports of ictal dancing or dancing-like movement. The previous reports provided either of description only or video only; or the case could hardly be called dancing. The “dancing epilepsy” was shown in a case of refractory complex partial seizures [1]. However, this article did not show the electroencephalography. Therefore, we cannot confirm whether the dancing was truly ictal or only a manifestation of post-ictal confusion. A report of ‘tap dancing in epilepsy’ showed a video with simultaneous electroencephalography in which rhythmic leg and foot tapping [2]. However, there was only slow wave activity, which indicates “post-ictal” dancing rather than “ictal”. In a recent report of a patient with left temporal lobe epilepsy [3], the authors provided a video with simultaneous electroencephalography but it could hardly be called dancing. Here, we present a case of dancing semiology in a patient with refractory temporal lobe epilepsy.

## Case presentation

**Fig. 1:**
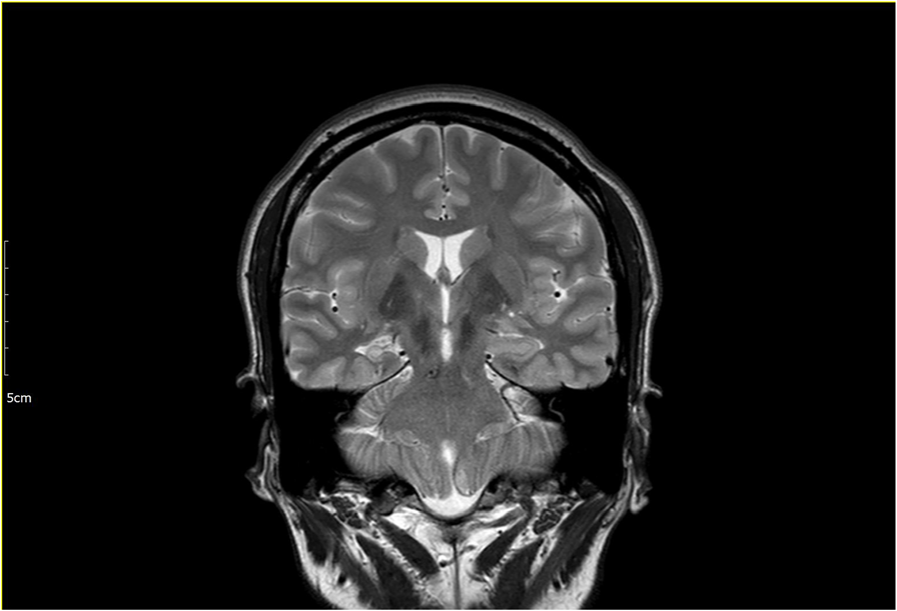
Magnetic Resonance Imaging. T2-weighted oblique coronal magnetic resonance imaging revealed right hippocampal atrophy


A 42-year-old right-handed woman suffered from weekly repetitions of unconscious dancing for 5 years, despite of multiple antiepileptic drugs including levetiracetam 3000 mg/d, valproate 900 mg/d, and pregabalin 300 mg/d. She was admitted to the epilepsy monitoring unit of a tertiary referral center for the feasibility of epilepsy surgery. On the initial clinical examination, she had no other symptoms or signs. The routine laboratory tests were negative. Magnetic resonance imaging showed right hippocampal atrophy (Fig. 1). Her habitual seizure was recorded during video-electroencephalography monitoring. The seizure began with the right hand automatism and ictal speech, which suggest that the ictal onset zone would be on the right side. An evolution of rhythmic delta activity was observed in the right temporal area beginning 16 s after the automatism (see Additional file 1: Video S1). As the ictal discharge spreads to the left temporal area, which means the secondary generalization, the ictal speech disappeared. After 20 s from the secondary generalization, she had rhythmical movement of her legs, similar to stepping through a dance, and the simultaneous video-electroencephalography showed regional slow waves over both of her frontal areas. When the ictal rhythm has switched to the left side, the left upper limb automatism and immobility of the right upper limb represented the rhythmic theta activity, which is still seen in the left temporal area. Taken together, we could identify the kicking and stepping like a dance as well as shaking left arm. According to her husband, the movement would have involved twirling dance, making a right turn when she was standing. However, it was not shown on the video. The dancing lasted even after the rhythmic discharge, which definitized the post-ictal dancing. We surmise that the “dancing” movement might be derived from some combination of automatism consisted of complex, rhythmic, and sequencial movement. She was completely amnestic with respect to the episode. Her ictal speech and the ictal electroencephalography imply that the right hippocampus atrophy should be the epileptogenic focus. There has been only one seizure for 3 months since a stereotactic gamma knife surgery applied to the atrophic right hippocampus.

Additional file 1:Simultaneous Video-electroencephalography Monitoring. Description of data: The seizure begins with right hand automatism and ictal speech, followed by an evolution of rhythmic delta activity in the right temporal area. After the secondary generalization, she showed rhythmical 2movement of legs, similar to stepping through a dance. Subsequent post-ictal paraphasia as well as confusion were documented. (WMV 12mb)


The authors declare that they adhered to CARE guidelines/methodology.

## Conclusions

This patient showed a semiology which can be classified as complex partial seizure followed by hypermotor seizure consisting of rhythmic movements of the extremities termed dancing [4]. Although it is difficult to distinguish dancing from hypermotor semiology, the former connotes more complex, rhythmical and sequencial movement of multiple body regions. Here, we report the most plausible dancing in the ictal and post-ictal state, documented by simultaneous video and electroencephalography. The underlying mechanism and symptomatogenic zone remain to be investigated.
